# Loneliness and its correlates among Bangladeshi older adults during the COVID-19 pandemic

**DOI:** 10.1038/s41598-022-19376-1

**Published:** 2022-09-02

**Authors:** Sabuj Kanti Mistry, A. R. M. Mehrab Ali, Uday Narayan Yadav, Md. Nazmul Huda, Saruna Ghimire, Manika Saha, Sneha Sarwar, Mark F. Harris

**Affiliations:** 1 ARCED Foundation, 13/1 Pallabi, Mirpur-12, Dhaka, Bangladesh; 2 Centre for Primary Health Care and Equity, University of New South Wales, Sydney, Australia; 3 BRAC James P Grant School of Public Health, BRAC University, Medona Tower, Bir Uttam AK Khandakar Road, Dhaka, 1213 Bangladesh; 4 National Centre for Epidemiology and Population Health, Research School of Population Health, The Australian National University, Canberra, Australia; 5 Translational Health Research Institute, School of Medicine, Western Sydney University, Campbeltown, NSW 2560 Australia; 6 Department of Sociology and Gerontology and Scripps Gerontology Center, Miami University, Oxford, OH USA; 7 Department of Human-Centred Computing, Faculty of Information Technology, Monash University, Clayton, VIC 3145 Australia; 8 Institute of Nutrition and Food Science, University of Dhaka, Dhaka, Bangladesh; 9 Department of Public Health, Daffodil International University, Dhaka, Bangladesh

**Keywords:** Epidemiology, Geriatrics

## Abstract

The present study aims to investigate the prevalence of loneliness and its associated factors among older adults during the COVID-19 pandemic in Bangladesh. This cross-sectional study was conducted in October 2020 among 1032 older Bangladeshi adults aged 60 years and above through telephone interviews. A semi-structured questionnaire was used to collect information on participants’ characteristics and COVID-19-related information. Meanwhile, the level of loneliness was measured using a 3-item UCLA Loneliness scale. More than half (51.5%) of the older adults experienced loneliness. We found that participants formally schooled [adjusted odds ratio (aOR = 0.62, 95% CI 0.43–0.88)] and received COVID-19-related information from health workers (aOR = 0.33, 95% CI 0.22–0.49) had lower odds of being lonely during the pandemic. However, older adults living alone (aOR: 2.57, 95% CI 1.34–4.94), residing distant from a health facility (aOR = 1.46, 95% CI 1.02–2.08) and in rural areas (aOR = 1.53, 95% CI 1.02–2.23) had higher odds of loneliness than their counterparts. Likewise, odds of loneliness were higher among those overwhelmed by COVID-19 (aOR = 1.93, 95% CI 1.29–2.86), who faced difficulty in earning (aOR = 1.77, 95% CI 1.18–2.67) and receiving routine medical care during pandemic (aOR = 2.94, 95% CI 1.78–4.87), and those perceiving requiring additional care during the pandemic (aOR = 6.01, 95% CI 3.80–9.49). The findings suggest that policies and plans should be directed to reduce loneliness among older adults who require additional care.

## Introduction

Loneliness is one of the most common psychological issues encountered by the older population worldwide^1^. Loneliness is an emotional and mental state^2^ that an individual faces in terms of subjective feelings of stress^3^, sadness, low self-esteem^4^, and hopelessness^5^. Globally, around one-third of the older adults reported being lonely^6^. A high level of loneliness has negative health consequences, including increased risk for heart disease, stroke, premature death, mental stress, chronic depression, dementia, and even a tendency to suicide^7–10^. Evidence suggests that the sense of loneliness raises stroke and dementia risk by 30% and 50%, respectively^11^. Among the older population (aged 60 years and above), loneliness can result in serious public health consequences, including increased hospital visits^12^, decreased quality of life^13^, and mortality^14^. According to a meta-analysis, among older adults, loneliness increases the risk of all-cause mortality by 26%^7^.

The older population is at increased risk for COVID-19 and associated physical and mental health consequences and deaths^15^. More than half of the COVID-19 deaths in China, Italy^16^, and India^17^, and almost 39% in Bangladesh^18^ were reported among the older population. They are also disproportionately affected by loneliness during this pandemic^19,20^. In line with this, recent evidence indicating a drastic increase in the global prevalence of mental health-related issues^21–23^. A longitudinal study conducted on US adults aged 50–80 years reported that the prevalence of isolation was more than doubled in 2020 compared to 2018 (56% vs. 27% in 2018)^24^. The most commonly reported factors associated with loneliness among older people include chronic diseases, retirement from work, staying alone away from children and family, widowhood, lack of social engagement and entertainment activities, sedentary lifestyle^25^, and physical disabilities^26,27^.

In Bangladesh, the first COVID-19 case was reported at the beginning of March 2020, and since then, the cases have constantly been increasing^28^. As of 13 July 2022, there were more than 1,992,058 confirmed COVID-19 cases with 29,217 deaths related to COVID-19 in Bangladesh^29^. The government of Bangladesh implemented restrictive measures such as lockdown and shutdowns^30^ to control the spread of the infection. While important to curb the spread of the infection, such measures also resulted in increased discomfort in getting everyday necessities and health care, in addition to creating an environment not conducive to mental health^28^. Some recent studies also documented increased mental health conditions such as stress, anxiety, fear, and depression among the older population in Bangladesh during the COVID-19 pandemic^31–34^. However, no study has explored the level of loneliness among the older population in Bangladesh during the COVID-19 pandemic, who are one of the most vulnerable population groups to the ongoing pandemic. Therefore, the present study aimed to investigate the prevalence of loneliness and its associated factors among older Bangladeshi adults amid the COVID-19 pandemic.

## Methods

### Study design and participants

This cross-sectional study was conducted remotely, through telephone interviews, in October 2020. Assuming a 50% prevalence of the outcome with a 5% margin of error, at 95% confidence level, 90% power of the test, and 80% response rate, the study sample size was estimated to be 1096. Although 1096 participants were approached, 1032 eligible participants agreed to participate, resulting in a ~ 94% response rate. A pre-existing registry, developed by the principal investigator’s institute, based on previously completed community-based studies, served as the sampling frame for the current study and included households from all eight administrative divisions of Bangladesh. To ensure representativeness from all eight divisions of Bangladesh, probability proportionate to the number of older adults in each division was used^35^. In each administrative division, households were selected using a simple random sampling technique, and subsequently, one eligible participant was interviewed from the selected households. Hence, the number of included households and respondents are equal. When a household had more than one eligible participant, the oldest member was selected for the interview. The only inclusion criterion was defined in terms of age (i.e., ≥ 60 years). The age limit was set to ≥ 60 years because the Government of Bangladesh identifies individuals aged 60 years and above as older adults^35^. Notably, the proportion of older adults in Bangladesh, currently 8% of the total population, is rapidly growing^35^ and is projected to increase to 21.9% by 2050^36^. The exclusion criteria included severe mental conditions (clinically diagnosed schizophrenia, bipolar mood disorder), a hearing disability, or an inability to communicate^28^.

### Data collection tools and techniques

A pre-tested semi-structured questionnaire was used to collect the information through a telephone interview. Data were collected electronically using SurveyCTO mobile app (https://www.surveycto.com/) and following the best practices for conducting phone surveys^37^. Our pre-existing registry, which had household contact information including mobile phone numbers, aided data collection. Participants were contacted on their mobile phones and interviewed by trained research assistants recruited based on previous experiences in administering health surveys on the electronic platform. The research assistants were trained extensively online in the Zoom platform before the data collection^28^. The interview sessions covered different methodological aspects of the study, tools and interviewing technique. The research assistants were trained on building rapport and instructed about the voluntary participation of the respondents and that they could skip any question they are not comfortable with. Participants who could not be reached on the first attempt were followed up multiple times and at different hours of the day. The English version of the questionnaire was first translated into Bengali language and then back-translated to English by two researchers to ensure the contents’ consistency. The questionnaire was then piloted among a small sample (n = 10) of older adults to refine the language in the final version. The tool used in the pilot study did not receive any corrections/suggestions from the participants in relation to the contents developed in the Bengali language. The interview was coundcted with this questionnaire which took around half an hour for each respondents. Considering the sensitivity of the topic we explored, the research assistants were also instructed to stop surveys and refer the respondents to the nearby primary care services if they felt stressed.

### Measures

#### Outcome measurement

The primary outcome of the study was loneliness, measured using a short 3-item UCLA Loneliness scale^38^. The three items included: how often do you feel (i) lack of companionship, (ii) left out, and (iii) isolated in the last two weeks. Each item in the scale was measured in terms of 3-item Likert responses: hardly ever (1 point), some of the time (2 points), and often (3 points). The participants were classified as lonely if they answered ‘some of the time’ or ‘often’ to any item^6^. Dichotomised loneliness variable was used for all data analyses. High internal consistency (Cronbach’s alpha 0.89) suggests the scale to be reliable among the study participants. A previous study has established the validity of the Bangladeshi version of the tool^39^.

#### Explanatory variables

We selected the explanatory variables based on the literature review^28,31^. Explanatory variables considered were administrative division (Barishal, Chattogram, Dhaka, Mymensingh, Khulna, Rajsahi, Rangpur, Sylhet), age in years (categorized as 60–69, 70–79, and ≥ 80), sex (male/female), marital status (married/without partner), family size i.e., number of individuals in the household (≤ 4 or > 4), family monthly income in Bangladeshi Taka (BDT) (< 5000, 5000–10,000, > 10,000), residence (urban/rural), occupation status (currently employed/unemployed and retired), formal schooling (yes/no), living arrangements (living alone or with family), health workers as the source of COVID-19 related information (yes/no), walking distance to the nearest health center (< 30 min/ ≥ 30 min), any prevalent noncommunicable conditions (NCDs) (yes/no), feeling concerned about COVID-19 (hardly, sometimes/often), feeling overwhelmed by COVID-19 (hardly, sometimes/often), difficulty earning and obtaining food, medicine, and routine medical care during the pandemic (hardly/sometimes to often), perception that older adults are highest risk of COVID-19 (yes/no), and required additional care during the pandemic (yes/no). Self-reported information on any prevalent medical conditions, such as arthritis, hypertension, heart diseases, stroke, hypercholesterolemia, diabetes, chronic respiratory diseases, chronic kidney disease, and cancer, was collected.

### Method of analysis

Descriptive analyses explored the distribution of variables in terms of frequencies and percentages. Chi-square tests compared the differences in loneliness by explanatory variables with a 5% significance level. Binary logistic regression models explored the factors associated with loneliness. The initial model was run with all potential covariates (listed in Table 1), and then, using the backward elimination criteria with the Akaike information criterion (AIC), the final model was selected^28^. The variables retained in the final model are presented in Table 2 and were adjusted for each other. Unadjusted and adjusted odds ratio (aOR) and associated 95% confidence interval (95% CI) are reported. All analyses were performed using the statistical software package Stata (Version 14.0).

**Table 1:** Participants’ characteristics and bivariate analyses (N = 1032).

Characteristics	Total	Experienced loneliness	Did not experience loneliness	*P*^a^
	n (%)	n (%)	n (%)	
Overall		531 (51.5)	501 (48.6)	
**Administrative division**				
Barishal	149 (14.4)	66 (44.3)	83 (55.7)	0.136
Chattogram	137 (13.3)	70 (51.1)	67 (48.9)	
Dhaka	210 (20.4)	120 (57.1)	90 (42.9)	
Mymensingh	63 (6.1)	29 (46.0)	34 (54.0)	
Khulna	158 (15.3)	83 (52.5)	75 (47.5)	
Rajshahi	103 (10.0)	51 (49.5)	52 (50.5)	
Rangpur	144 (14.0)	83 (57.6)	61 (42.4)	
Sylhet	68 (6.5)	29 (42.7)	39 (57.4)	
**Age (year, %)**				
60–69	803 (77.8)	399 (49.7)	404 (50.3)	0.102
70–79	174 (16.9)	101 (58.1)	73 (42.0)	
≥ 80	55 (5.3)	31 (56.4)	24 (43.6)	
**Sex**				
Male	676 (65.5)	328 (48.5)	348 (51.5)	0.009
Female	356 (34.5)	203 (57.0)	153 (43.0)	
**Marital status**				
Married	840 (81.4)	427 (50.8)	413 (49.2)	0.404
Without partner^b^	192 (18.6)	104 (54.2)	88 (45.8)	
**Family size**				
≤ 4	318 (30.8)	165 (51.9)	153 (48.1)	0.853
> 4	714 (69.2)	366 (51.3)	348 (48.7)	
**Family monthly income (BDT)**^**c**^				
< 5000	145 (14.1)	96 (66.2)	49 (33.8)	< 0.001
5000–10,000	331 (32.1)	149 (45.0)	182 (55.0)	
> 10,000	556 (53.8)	286 (51.4)	270 (48.6)	
**Residence**				
Urban	269 (26.1)	123 (45.7)	146 (54.3)	0.029
Rural	763 (73.9)	408 (53.5)	355 (46.5)	
**Occupation status**				
Currently employed	419 (40.6)	225 (53.7)	194 (46.3)	0.233
Unemployed/retired	613 (59.4)	306 (49.9)	307 (50.1)	
**Formal schooling**				
No	602 (58.3)	330 (54.8)	272 (45.2)	0.011
Yes	430 (41.7)	201 (46.7)	229 (53.3)	
**Living arrangement**				
Living with family	953 (92.3)	474 (49.7)	479 (50.3)	< 0.001
Living alone	79 (7.7)	57 (72.2)	22 (27.9)	
**Health workers as the source of COVID-19 information**				
No	297 (28.8)	137 (46.1)	160 (53.9)	0.021
Yes	735 (71.2)	337 (45.9)	398 (54.2)	
**Walking distance to the nearest health centre**				
< 30 min	508 (49.2)	239 (47.1)	269 (53.0)	0.005
≥ 30 min	524 (50.8)	292 (55.7)	232 (44.3)	
**Feeling concerned about COVID-19**				
Hardly	299 (29.0)	94 (31.4)	205 (68.6)	< 0.001
Sometimes to often	733 (71.0)	437 (59.6)	296 (40.4)	
**Feeling overwhelmed by COVID-19**				
Hardly	370 (36.4)	118 (31.9)	252 (68.1)	< 0.001
Sometimes to often	647 (63.6)	402 (62.1)	245 (37.9)	
**Any prevalent chronic noncommunicable condition**				
No	424 (41.1)	173 (40.8)	251 (59.2)	< 0.001
Yes	608 (58.9)	358 (58.9)	250 (41.1)	
**Perceived that older adults are at the highest risk of COVID-19**				
No	416 (41.1)	188 (45.2)	228 (54.8)	0.001
Yes	616 (58.9)	343 (55.7)	273 (44.3)	
**Difficulty earning during the pandemic**				
No difficulty	340 (37.4)	98 (28.8)	242 (71.2)	< 0.001
Difficulties faced	570 (62.6)	364 (63.9)	206 (36.1)	
**Difficulty procuring food during the pandemic**				
No difficulty	553 (55.3)	213 (38.5)	340 (61.5)	< 0.001
Difficulties faced	447 (44.7)	292 (63.3)	155 (34.7)	
**Difficulty obtaining medicine during the pandemic**				
No difficulty	733 (75.3)	323 (44.1)	410 (55.9)	< 0.001
Difficulties faced	240 (24.7)	169 (70.4)	71 (29.6)	
**Difficulty receiving routine medical care during the pandemic**				
No difficulty	644 (69.6)	258 (40.1)	386 (59.9)	< 0.001
Difficulties faced	281 (30.4)	214 (76.2)	67 (23.8)	
**Perceived needing additional care during the pandemic**				
No	769 (74.5)	312 (40.6)	457 (59.4)	< 0.001
Yes	263 (25.5)	219 (83.3)	44 (16.7)	

^a^*P* value obtained from Chi-square test evaluating the differences between those who experienced and did not experience loneliness.

^b^Without partner group includes divorced, separated and never married.

^c^BDT stands for Bangladesh taka and 1 BDT ~ 84.7 US dollars.

**Table 2:** Factors associated with loneliness among the participants (N = 1032).

Characteristics	cOR^a^	95% CI	*P*	aOR^b^	95% CI	*P*
**Family size**						
≤ 4	Reference			Reference		
> 4	0.98	0.75–1.27	0.853	0.75	0.52–1.09	0.130
**Residence**						
Urban	Reference			Reference		
Rural	1.36	1.03–1.80	0.029	1.53	1.02–2.23	0.040
**Occupation status**						
Currently employed	Reference			Reference		
Unemployed/retired	0.86	0.67–1.10	0.233	0.76	0.53–1.08	0.496
**Formal schooling**						
No	Reference			Reference		
Yes	0.72	0.56–0.93	0.011	0.62	0.43–0.88	0.008
**Living arrangement**						
Living with family	Reference			Reference		
Living alone	2.62	1.58–4.35	< 0.001	2.57	1.34–4.94	0.005
**Health workers as the source of COVID-19 related information**						
No	Reference			Reference		
Yes	0.45	0.34–0.59	< 0.001	0.33	0.22–0.49	< 0.001
**Walking distance to the nearest health centre**						
< 30 min	Reference			Reference		
≥ 30 min	1.41	1.11–1.81	< 0.001	1.46	1.02–2.08	0.040
**Feeling overwhelmed by COVID-19**						
Hardly	Reference			Reference		
Sometimes to often	3.50	2.67–4.59	< 0.001	1.93	1.29–2.86	0.001
**Any prevalent chronic noncommunicable condition**						
No	Reference			Reference		
Yes	2.08	1.61–2.67	< 0.001	1.28	0.88–1.87	0.197
**Difficulty earning during the pandemic**						
No difficulty	Reference			Reference		
Difficulties faced	4.35	3.26–5.83	< 0.001	1.77	1.18–2.67	0.006
**Difficulty obtaining medicine during the pandemic**						
No difficulty	Reference			Reference		
Difficulties faced	3.02	2.21–4.13	< 0.001	1.36	0.82–2.26	0.234
**Difficulty receiving routine medical care during the pandemic**						
No difficulty	Reference			Reference		
Difficulties faced	4.78	3.48–6.56	< 0.001	2.94	1.78–4.87	< 0.001
**Perceived that they required additional care during the pandemic**						
No	Reference			Reference		
Yes	7.29	5.12–10.39	< 0.001	6.01	3.80–9.49	< 0.001

^a^Crude odds ratio.

^b^Adjusted odds ratio (adjusted for all the variables shown in this table). The initial model was run with all potential covariates listed in Table 1. Then, using the backward elimination criteria with the Akaike information criterion (AIC), the final model with variables listed in this table was selected.

### Ethics approval

The institutional review board of the Institute of Health Economics, University of Dhaka, Bangladesh, approved the study protocol (Ref: IHE/2020/1037), and the guidelines of the Declaration of Helsinki were followed in every stage of the study. Verbal informed consent was sought from the participants before administering the survey. Participation was voluntary, and participants did not receive any compensation^28^.

## Results

### Characteristics of the participants

Table 1 shows the summary statistics of the study participants. Among the 1032 study participants, 20.4% were from the Dhaka division, 77.8% were aged 60–69 years, 65.5% were male, 81.4% were currently married, 73.9% were rural residents, and 58.3% lacked formal schooling. Over half of the participants (53.8%) had a family income of > 10,000 BDT, 40.6% were currently employed, 92.3% resided with family members, and 58.9% had at least one pre-existing NCD. Moreover, 71.0% of the participants were concerned about the pandemic, 62.6% had difficulty earning, 24.7% had difficulty procuring medicine, and 30.4% had difficulty receiving routine medical care during the pandemic (Table 1).

### Prevalence of loneliness

Overall, more than half (51.5%) of the participants experienced loneliness (Table 1). In bivariate analyses, loneliness was significantly higher among females (57.0%), those in the lowest income bracket (66.2%), rural residents (53.5%), those without formal schooling (54.8%), living alone (72.2%), and with prevalent NCDs (58.9%). The prevalence of loneliness was also higher among participants concerned about (59.6%) and overwhelmed by the pandemic (62.1%), and those perceiving that older adults are at the highest risk (55.7%) and requiring additional care during the pandemic (83.3%). Moreover, loneliness was significantly higher among participants facing difficulty earning (63.9%) and obtaining food (63.3%), medicines (70.4%), and routine medical care (76.2%) during the pandemic. Meanwhile, loneliness was significantly lower among those receiving COVID-19 information from health workers (45.9%) (Table 1).

### Factors associated with loneliness

Table 2 shows the factors associated with loneliness in the final adjusted model. In the adjusted model, loneliness was associated with formal schooling, rural residence, living arrangements, health workers as the source of COVID-19 information, walking distance to the nearest health centre, feeling overwhelmed by COVID-19, perceived difficulty in earning and obtaining medical care, and perceived need of additional care during the pandemic. We found that participants formally schooled had nearly 40% lower odds of being lonely than those without formal schooling (aOR = 0.62, 95% CI 0.43–0.88). Similarly, participants receiving COVID-19-related information from health workers (aOR = 0.33, 95% CI 0.22–0.49) had lower odds of being lonely during the pandemic. Compared to participants living with family, those living alone were 2.5 times more likely to experience loneliness (aOR: 2.57, 95% CI 1.34–4.94). Likewise, participants living distant (≥ 30 min walking) from a health facility (aOR = 1.46, 95% CI 1.02–2.08), residing in rural areas (aOR = 1.53, 95% CI 1.02–2.23), overwhelmed by COVID-19 (aOR = 1.93, 95% CI 1.29–2.86), and facing difficulty in earning (aOR = 1.77, 95% CI 1.18–2.67) and receiving routine medical care (aOR = 2.94, 95% CI 1.78–4.87) had higher odds of loneliness than their counterparts. Moreover, the participants who perceived that they required additional care during the pandemic had more than five times higher odds of loneliness (aOR = 6.01, 95% CI 3.80–9.49) than those who did not feel so.

## Discussion

This study investigated the prevalence of loneliness and its correlates among Bangladeshi older adults during the COVID-19 pandemic. More than half of the participants experienced loneliness during the COVID-19 pandemic. We did not find any single study on older adults’ loneliness during the pandemic in Bangladesh and other low- and middle-income countries to compare the current study’s findings. However, an online self-reporting survey on the general Bangladeshi population (aged ≥ 15) found a higher prevalence of loneliness (71%) than the current study^40^. While, another study conducted among graduate students in Bangladesh reported a slightly lower prevalence of loneliness (43.3%) during the pandemic^41^. The plausible reasons for such a difference in loneliness prevalence include differences in study tools to measure loneliness, differences in the age range of participants, and online mode of survey administration. The prevalence of loneliness (52%) in the current study is similar to a previous study from the US (54%)^42^, but higher than other studies from the US^43^, the United Kingdom^44^, and Canada^15^, which reported prevalence ranging from 30 to 43%. Several factors, including study design, sampling differences, and socioeconomic contexts, may explain the variations in individuals’ loneliness during the pandemic. The current study contributed to the limited international literature^15,42–44^ that has examined loneliness prevalence among older adults and its predictors during the COVID-19 pandemic.

The high prevalence of loneliness among older Bangladeshi adults was expected, given their high age-related changes and losses such as loss of jobs (due to retirement), partners, friends, and social networks (due to death)^45,46^. Declining physical and cognitive health also makes older adults more vulnerable to loneliness^47^. Further, social interaction and participation, two important protective factors of loneliness^48^, were minimized amid the pandemic. Due to COVID-19-related lockdown and isolation measures, older adults’ infrequent interactions with relatives, neighbours, and friends^49^, inadequate social participation in voluntary and religious activities^50^, and limited regular activities^51,52^ may result an increase in loneliness. Our study’s findings highlight the necessity for undertaking interventions to engage older adults in activities and improve social interactions and community participation (while practising safety measures to curb the COVID-19 spread) to decrease the likelihood of experiencing loneliness.

Our study revealed that, older individuals with formal education had lower odds of being lonely during the COVID-19 pandemic. This finding is similar to previous literature, which identified higher education as an essential correlate of loneliness^53,54^. This could be because educated people may be aware of the adverse physical and mental health consequences of loneliness and its coping strategies^55^. Neuroticism and stress are directly related to loneliness, and educational attainment reduces loneliness by decreasing the vulnerability to neuroticism and stress^56^. Furthermore, education has been considered a proxy for socioeconomic status and correlates with income and accessibility to resources^57^. Education can reduce loneliness by enriching social networks and connectedness with friends and external individuals via social media^58^. In contrast, illiteracy restricts access to information on community events and resources, limiting social activity and participation^59^, thus increasing the likelihood of loneliness. This suggests that education enhances the social activity and community participation and decreases loneliness in older adults.

The current study suggested that female participants and those living in rural areas were more likely to feel lonely than males and their urban counterparts, similar to existing literature^46,60^. Our finding that older adults who lived alone (without family) were more like to feel lonely is supported by 15 studies included in a review^26^ and several other studies^43,61,62^. As indicated above, older individuals may live alone due to their loss of spouses and limited relationships with their family members^45,46^. Specifically, older females are more likely to be alone due to higher life expectancy and increased likelihood of widowhood, making them vulnerable to chronic diseases, and poor functional status in later life^60^. Household members are the first line of social networks and provide inherent opportunities for socialization which otherwise may be unavailable to those living alone, leading to more loneliness^26^. Older rural residents, especially females, may experience many challenges^11^, including limited transportation facilities, inadequate financial means, and reduced access to internet services^11,63^. Such limited facilities tend to isolate them from family and community members, thus increasing their risk of loneliness^63^. Loneliness in elderly women may be compounded by lack of financial resources, supports and access to health care especially in rural areas^64^. Despite this, there are no loneliness prevention interventions for older adults in Bangladesh^65^. Our findings suggest undertaking loneliness prevention interventions for older people, specifically for females and rural residents.

Our study also indicated that older individuals who received COVID-19-related information from healthcare workers had lower odds of being lonely during the pandemic. To the best of our knowledge, this is the first study investigating the association between loneliness in older adults and the source of COVID-19-related information, such as healthcare workers. For those individuals, in addition to COVID-19-related information, healthcare workers may have provided additional information on health and well-being, such as the negative consequences of loneliness on their mental health and strategies to cope with it^66^. Such awareness of the negative aspects of loneliness may prompt individuals to take action to decrease loneliness^67^. Our study’s findings highlight that it is vital to disseminate information about reducing loneliness during the pandemic alongside COVID-19-related information.

Several COVID-19 pandemic-related measures, such as feeling overwhelmed by the pandemic, difficulty earning during the lockdown and receiving routine medical care, and perception that older people required additional care during the pandemic, were associated with an increased likelihood of experiencing loneliness. To our knowledge, this is the first study exploring the associations of these correlates with loneliness among older adults during the pandemic. The government of Bangladesh has taken some strong measures to control the spread of COVID-19^68,69^. However, it has not prioritized the mental, financial, and social wellbeing of its people, specifically older adults^33^. Thus, the findings could be explained on the grounds of psychological distress due to the ongoing pandemic^70^. On the one hand, as previously mentioned, the advent of COVID-19 has reduced visits of family members/friends and emotional closeness^1^, disrupted and overwhelmed older adults’ lives^31,71^. These COVID-19-related changes in the daily lives increased tension, anxiety, fear, and the risk of developing loneliness among older adults^1^. On the other hand, frustration was induced in terms of the inability to meet daily needs, make earnings, and access health care during the lockdowns^33^. Poor income is the strongest predictor of loneliness^26^. Furthermore, inaccessibility to materialistic and financial resources increases loneliness through low self-esteem and self-efficacy^47^. As part of the nationwide lockdown in Bangladesh, public transportation was restricted to limit mass movement^69^. Given that most Bangladeshi population rely on public transit, such a halt of transportation services meant no vehicular means to go to jobs or health facilities. Closure to business meant job loss, either for older adults themselves or their family members they were directly dependent on^72^. In the absence of financial aid from the government, such economic loss may have brought financial distress to the family^73^. Participants in the current study who perceived that they required additional care during the pandemic were more than five folds more likely to experience loneliness. The absence of targeted policies to address their needs during these crucial times may have caused additional distress as they may have felt ignored, left out, and lonely. Therefore, the current study findings highlight the importance of providing additional support and care, including economic and mental health support, during public health emergencies.

## Strengths and limitations of the study

Our study has several strengths. First, this study is among the first in the literature from Bangladesh to examine the prevalence of loneliness and its correlates among Bangladeshi older adults during the COVID-19 pandemic. Second, to the best of our knowledge, some of the correlates of loneliness in older adults (e.g., receiving COVID-19-related information from healthcare workers, feeling overwhelmed by the COVID-19 pandemic, perceiving that they required additional care during the pandemic, difficulty receiving routine medical care during the pandemic, and distance to the nearest healthcare centre) in the current study have been reported for the first time in Bangladesh and globally. Despite these strengths, our study’s findings should be considered in the context of its limitations. First, our research was cross-sectional in nature. Therefore, causality cannot be established. Second, amidst the pandemic, we had to conduct telephone interviews, and it is likely that the sample may not be representative of the entire older population of Bangladesh, specifically excluding those who don’t have telephone access. Third, our study is limited to quantitative analysis, as we did not explore the qualitative aspects of older adults’ feelings of loneliness during the pandemic. These limitations highlight the need for further studies with a mixed-method approach, including a qualitative study exploring older adults’ experience of loneliness and its associated factors during the COVID-19 pandemic. This will provide a better understanding of older adults’ feelings of loneliness and the related factors during the COVID-19 pandemic in Bangladesh.

## Conclusion

The present study revealed that a high proportion of older adults experienced loneliness during this COVID-19 pandemic in Bangladesh and suggests the need for supportive mental health intervention focusing on this vulnerable population. It is also very important to address various factors associated with loneliness identified in this study by providing information and improving access to health care. Policymakers and health care practitioners should also consider strengthening the social support structure for the older population as part of the emergency management plan, and involving health workers can be of value in this regard.

## Data Availability

Data is available upon reasonable request to the corresponding author.
